# Changes in intensification of religious involvement during the COVID-19 pandemic in Poland

**DOI:** 10.1371/journal.pone.0269015

**Published:** 2022-06-15

**Authors:** Rafał Boguszewski, Marta Makowska, Monika Podkowińska

**Affiliations:** Institute of Sociological Sciences and Pedagogy, Warsaw University of Life Sciences, Warsaw, Poland; Boston Children’s Hospital, UNITED STATES

## Abstract

**Introduction:**

The emergence of the virus Sars-CoV-2, and subsequent COVID-19 pandemic, caused a global threat to public health. In such challenging and worrying situations it can be expected that people will seek comfort in religion. However, before the pandemic there were never such wide world disruptions of religious practice–because of social distancing regulations, religiosity cannot be practiced in the same way as it was before the pandemic.

**Methods:**

Two anonymous online surveys were conducted of adult Poles, one during the second wave (N = 1072; November 2020) and one during the third wave of the pandemic (N = 1080; April 2021). The survey samples of participants reflected the Polish population structure in terms of age, sex, size of place of residence, education, and province.

**Results:**

Participation in institutional religious practices fell threefold to 11.9% during the pandemic, as compared to pre-pandemic times (31.2%). The number of people who did not go to church at all increased from 23.1% to 57.0%. Between the second and third waves, there was a small return to regular practice (from 10.1% to 14.1%). Based on the subjective assessment of the amount of time devoted to prayer, fewer people reporting an increase (10.6%) than a decrease (20.1%) in religious commitment during the pandemic. Logistic regressions showed that an increase in religious commitment can be predicted by older age, more frequent participation in religious practices before the pandemic, and higher life satisfaction. A decrease in religious commitment can be predicted by younger age, less frequent participation in religious practices before the pandemic, and greater number of children in the household.

**Conclusion:**

Our research indicates a deintensification of religious practices during the COVID-19 pandemic in Poland. The pandemic has accelerated process of polarization of Polish religiosity.

## Introduction

Socio-cultural changes, related to the transformation from tradition to modernity, lead to secularization and atheization of society, whereby ties with religion and the Church are severed, and social structures are freed from the control of religious institutions [1, 2]. Socio-cultural changes can also lead to privatization of religion. Privatization is a term that can be understood as: religion without the churches; churches as voluntary organizations; individual theological responsibility; religious subjectivism; separation of religion from public concerns; the religious marketplace [3]. As such, religion does not disappear, as it does in the secularization paradigm, but it becomes a matter of preference and choice, while losing overall normative power [4, 5].

On the other hand, there are concepts indicated on the phenomenon of desecularization contradicts that of the disappearance of religion, and suggests a continuing strong presence of religion in the world [6–8]. Karpov [9] claims that first is secularization and then is desecularization and this phenomenon does not apply to societies where religion lasts all of the time. Religiousness in the postmodern world should be assessed primarily as a function of the sudden changes, instability, and differentiation taking place in it. It is assumed that traditional religions are weakening, but a religion in its new forms maintains an important position, providing people with answers to difficult questions about the essence and meaning of life, for which rationalism is often ineffective.

There is no doubt that Europe has been experiencing a deviation from traditional religiosity for some time, although process varies from country to country [6, 10, 11]. The transformation in religiosity has also affected Poland, though are not happening as fast as in other countries, for example in Ireland [12]. The European Social Survey shows that, among countries that are predominantly Catholic, Poland has historically had the highest percentage of people who consider themselves adherents to a certain religion, though this fell from 93.3% in 2002 to 87.2% in 2018 [12]. Sociological studies on religiosity also suggest that there has been a systematic decline in religious practices in Poland over the last 20 years [12–14]. According to data from the Institute of Statistics of the Catholic Church, the percent of *dominicantes* (meaning congregation or people participating in Sunday mass) fell from 45.2% in 2002 to 36.9% in 2019 [14]. This decline is permanent and noticeable [14]. The gradual secularization of Polish society is explained, *inter alia*, by the integration into new Europe [6]; the fact that traditional values lose their importance in favor of modern ones [15]; the end of the pontificate of John Paul II, who was a religious and moral authority for many Poles [15, 16]; changes in the perception of the moral authority of the Church [15]; loss of confidence in the Church after exposure of the child abuse scandals [12, 17]; political connections of the Church and its involvement in current political disputes [15].

There is no doubt that after a long period of stabilization, religiosity in Poland has changed in recent years. Precisely how this change was influenced by the COVID-19 pandemic, which impacted most dimensions of social life, including the religious dimension, remains unclear.

During the COVID-19 pandemic, Polish society, like many others, was in an unprecedented public health emergency. It was reasonable to assume that many people sought consolation in God, given the above-average religiousness of society. Such a situation aligns with the classical sociological theories that people seek solace in religion [18, 19]. Currently published papers also underline the importance of religion and religious capital in dealing with the experienced threat and maintaining well-being during the Covid-19 pandemic [20–24]. Research conducted during the pandemic confirm these claims, indicating that religious coping in crisis was a mechanism used [23, 25–27]. However, some publications suggest that the pandemic–along with the accompanying restriction and limited possibility of participating in institutional religious cults–led to a decline in religious commitment among the faithful [28–30].

In Poland pandemic-associated governmental restrictions also applied to churches, but at no stage were restrictions as strict as those of some neighboring countries e.g., Germany, where private prayer in places of public worship was closed and public religious gatherings were forbidden [31]. Table 1 presents the epidemiological situation and restrictions enforced in Poland during particular periods of the COVID-19 pandemic. The restrictions did not change as dynamically during the second and third waves as during the first. There is no doubt, however, that the pandemic-related social restrictions significantly influenced church functions and the implementation of the mission. Thus, changes caused by the pandemic primarily concerned institutional religion, for example, the shift of many religious practices to the Internet and acceleration of the medialization of religion [32]. These changes certainly influenced the religiosity of Poles, which thus far was primarily traditional.

**Table 1:** Timeline of restrictions in Poland.

First wave							Second wave			Third wave	
Date	13 March 2020	24 March 2020	15 April 2020	19 April 2020	16 May 2020	29 May 2020	9 October 2020	16 October 2020	6 November 2020	19 March 2021	11 June 2021
**Total cases**	68	901	7,582	9,287	18,257	23,155	116,338	157,608	493,765	2,010,295	2,877,004
**Daily new cases**	17	152	380	545	241	330	4,739	7,705	27,086	26,049	338
**Total coronavirus deaths in Poland**	2	10	286	360	915	1,051	2,919	3,440	7,286	48,810	74,515
**Restrictions**	Max. 50 people can participate in religious rituals.	Max. 5 people can participate in religious rituals.	From April 16, the obligation to cover the mouth and nose in the church (not for the worshiper) was introduced.	From April 20 max. 1 person per 15 m^2^ in churches. Mouth and nose have to be covered.	From May 17 max. 1 person per 10 m^2^ in churches. Mouth and nose have to be covered.	Removal of restrictions, except obligation to cover mouth and nose. 2 m. distance between the faithful.	Poland is divided into 3 zones: red (highest number of cases), yellow (medium number of cases) and green (low number of cases). In red: 50% of church occupancy. In yellow: 1.5 m. distance between the faithful. Mouth and nose have to be covered.	In red: max. 1 person per 7 m^2^ in churches. 1.5 m. distance between the faithful. In yellow: max. 1 person per 4 m^2^ in churches. 1.5 m. distance between the faithful. Mouth and nose have to be covered.	Whole Poland is red zone since 24 October. Max. 1 person per 15 m^2^ in churches. 1.5 m. distance between the faithful. Mouth and nose have to be covered. This regulation is prolonged by next regulations.	Max. 1 person per 15 m^2^ in churches. 1.5 m. distance between the faithful. Mouth and nose have to be covered. This regulation is prolonged by next regulations.	From June 13 50% of church occupancy. 1.5 m. distance between the faithful. From June 26 75% of church occupancy. 1.5 m. distance between the faithful. Mouth and nose have to be covered.

Based on: [33–43].

Religiosity can be defined in various ways and its various dimensions are indicated such as intellectual, ideological, ritualistic, experiential, and consequential [44, 45]. Sociological research on religiosity is most often based on the ritualistic dimension (religious practices). Our research also approaches religiosity in such a narrow sense of religious practices, but we are aware that it is quite a simplification. However, religious practices are defined herein not only as institutional religiosity but also religious commitment, expressed in subjective assessment of the amount of time devoted to prayer, meditation, and other religious practices.

This study aims to describe the changes in the religious practices of Poles during the COVID-19 pandemic. Therefore, we asked the following research questions: 1) How did the pandemic affect institutional religiosity? 2) Has the religious commitment, expressed in subjective assessment of the amount of time devoted to prayer, meditation, and other religious practices, changed during the pandemic? 3) Is it possible to identify any socio-demographic features that can predict an increase or a decrease in religious commitment during the pandemic?

The fundamental research hypothesis is based on classical sociological theories [18, 19] and the results of some studies from the beginning of the pandemic [23, 25–27], “It is likely that during the pandemic, more people have increased their religious commitment, expressed in subjective assessment of the amount of time devoted to prayer, meditation and other religious practices than have reduced it, because people in crisis situations seek solace in religion.”

## Methods

The research project on which this article is based has addressed various social aspects of the pandemic. So far, four online surveys have been carried out under this project 1) during the first wave of the pandemic; 2) after the end of the first wave; 3) during the second wave of the pandemic; 4) during the third wave of the pandemic. In this article, we will focus only on the last two, as they expanded the issue of religiosity–the third survey which was carried out on 24–27 November 2020 (N = 1072), and the fourth research carried out on 26–28 April 2021 (N = 1080). At the time of both studies there was a lockdown in Poland.

Sample sizes were estimated based on those that would be reasonable for independent samples *t-*tests, and calculations were carried out using G*Power 3.1 [46]. Around 20 million adult Poles have Internet access, assuming an alpha of 0.05, desired power of 0.80, a (small) effect size of *d* = 0.2, and an allocation ratio of 0.25, the necessary sample size was shown to be 968 respondents. An allocation ratio of 0.25 was used to allow for substantial differences in frequencies of category membership for the various socio-demographic characteristics considered in the studies. Hence, we gathered data from more than 1,000 respondents in each study.

The selection of the respondents in both analyzed surveys were conducted through stratified quota sampling of an internet panel (SW Panel—one of the largest internet panels in Poland). The respondent samples represent a cross-section of Polish societal structure in terms of gender (2 groups), age (5 groups), size of place of residence (4 groups), province (16 groups) and education (2 groups: higher and other). Data from surveys and the survey questionnaires are public and available on figshare (https://figshare.com/articles/dataset/Poland_Covid-19_3rd/14398835 and https://figshare.com/articles/dataset/Poland_Covid-19_Fourth_Wave/16566612/2).

The surveys were anonymous, the respondents took part voluntarily and could withdraw at any time. Participants for completing the survey received some points that can be exchanged for prizes in the rewards pool. At the time of the study there was no Ethics Committee for Social Science at Warsaw University of Life Sciences. However, an external company (SW Research) commissioned by Warsaw University of Life Sciences undergoes an annual quality audit and had a current Organization of Opinion and Market Research Firms (OFBOR) certificate at the time of the study, which guarantees compliance with all applicable standards in the field of quantitative research. Polish authorities do not require ethics committee consent for online surveys carried out anonymously with consenting adult respondents.

Data were analyzed using IBM SPSS Statistics software (version 27.0). Data analysis was based primarily on frequency distribution and cross-tabulation. For the purposes of further analysis, we have aggregated the data from the two surveys. The people who participated in both surveys (N = 185) were included only once, using responses from the first survey (24–27 November 2020). The aforementioned aggregation gives the sample of 1967 respondents. The aggregated set allowed for further comparisons, but above all as a result of this operation, the numbers of people who declared both an increase and a decrease in their religious commitment (expressed in subjective assessment of the amount of time devoted to prayer) during the pandemic were numerous enough to be able to do the logistic regressions and talk more about socio-demographic structure of each of those two groups.

The first logistic regression predicts an increase in a religious commitment based on respondents’ socio-demographic characteristics, where 1 = people who declared that they spend more time (in a pandemic) on prayer, meditation, and other religious practices than before the pandemic (N = 147) and 0 = others (N = 1614). The second logistic regression predicts a decrease in a religious commitment based on respondents’ socio-demographic characteristics, where 1 = people who declared that they spend less time (in a pandemic) on prayer, meditation, and other religious practices than before the pandemic (N = 280) and 0 = others (N = 1481). Crosstables were done between religiosity before the pandemic and religious commitment during the pandemic. Chi-square tests and Cramer’s *V* coefficients were used.

## Results

Data indicate that regular participation in institutional practices (institutional religiosity) during the pandemic decreased almost threefold from 31.2% to 11.9%, compared to declared participation in institutional practices before the pandemic (Table 2). Concurrently, the percentage of people who do not practice religion in church increased from 23.1% before the pandemic to 57.0% during the pandemic (Table 2). The number of non-regular practitioners remained almost unchanged at 13.1% before and 12.9% during the pandemic. A slightly larger difference occurred among those who practiced occasionally, at 23.7% before the pandemic and 18.1% during. The return to regular religious practice slightly increased (from 10.1% to 14.1%) between the second and third waves of the pandemic. The number of non-regular and occasional religious practitioners also increased from 11.5% to 14.2% and from 16.9% to 19.8%, respectively (Table 2).

**Table 2:** Participation in religious services held in a church.

During the pandemic, have you sometimes participated in masses and other religious services held in a church?	2nd pandemic wave (24.11–27.11.2020)	3rd pandemic wave (26.04–28.04.2021)	Declared participation in institutional practices during the pandemic*	Declared participation in institutional practices before the pandemic *
	N = 1072%/(n)	N = 1080%/(n)	N = 1967%/(n)	N = 1967%/(n)
Yes, regularly (at least once a week)	10.1% (108)	14.1% (152)	11.9% (234)	31.2% (613)
Yes, but not regularly (1–2 times a month)	11.5% (123)	14.2% (153)	12.9% (254)	13.1% (257)
Yes, but occasionally (less than once a month)	16.9% (181)	19.8% (214)	18.1% (357)	32.7% (643)
No	61.6% (660)	51.9% (561)	57.0% (1122)	23.1% (454)

* Based on the aggregated surveys.

During the pandemic, approximately one in ten Poles (10.6%) spent more time in prayer, meditation and other religious practices than before, however, almost twice as many (20.1%) spent less time. The majority of people (43.5%) claimed that time for prayer, meditation and other religious practices remained unchanged during the pandemic. These opinions did not change significantly between the second wave of the pandemic and the third (Table 3).

**Table 3:** Religious commitment during the pandemic.

Has your religious commitment changed during the coronavirus pandemic? *	2nd pandemic wave (24.11–27.11.2020)	3rd pandemic wave (26.04–28.04.2021)	Aggregated surveys
	N = 726%/(n)	N = 664%/(n)	N = 1390%/(n)
Yes, nowadays I spend more time in prayer, meditation and other religious practices	9.1% (66)	11.7% (81)	10.6% (147)
Yes, I spend less time now praying, meditating and other religious practices	19.0% (138)	20.6% (142)	20.1% (280)
No, I spend the same amount of time in prayer, meditation and other religious practices	45.3% (329)	42.5% (275)	43.5% (604)
Hard to say	26.6% (193)	25.2% (166)	25.8% (359)

*The answer “Not applicable. I do not practice and have not practiced religion” was omitted.

Two logistic regression analyses were performed to test whether religious commitment during the pandemic could be predicted from socio-demographic characteristics (see Tables 3 and 4). Religious commitment significantly increased with the age increase, participation in religious practices increased (defined as the frequency of participation in religious masses, services, and meetings before the introduction of social isolation during the pandemic) and life satisfaction increased. Gender, population size of place of residence, education, number of children, self-assessments of health, financial situation or COVID-19 infection of the respondent, or any of family or close friends, were not significant predictors of increase in religious commitment (Table 4). The created model as a whole was better than a constant only model, χ^2^(10) = 162.242, *p* < 0.001, Nagelkerke *R*^2^ = 0.224.

**Table 4:** The logistic regression analysis predicting increase in religious commitment based on respondents’ socio-demographic characteristics (N = 1761*).

Predictor	B	SE B	p	Exp(B)
Gender (female-male)	.052	.199	.793	1.053
Age group (increasing)	.229	.080	**.004**	1.258
Population size of place of residence (increasing)	-.038	.072	.601	.963
Education (increasing)	-.108	.084	.199	.898
Number of children (under 18 years of age) living in household (increasing)	.147	.112	.188	1.159
Self-assessment of health (increasing)	.203	.158	.201	1.224
Financial situation (increasing)	.030	.166	.855	.970
Participation in religious practices when not socially isolating (increasing)	.885	.097	**.000**	.413
Respondent or any of his/her family or close friends infected with coronavirus (no–yes)	-.293	.204	.150	1.341
Life satisfaction (increasing)	.357	.122	**.003**	.700
Constant	.108	.782	.890	.897

Criterion variable: declared increase of religious commitment (0 = No, 1 = Yes). Aggregated surveys. Bold indicates were is statistical significance.

* People who chose the answer ‘Not applicable. I do not practice and have not practiced religion’ were excluded from the analysis.

Religious commitment significantly decreased with the age decrease, participation in religious practices decreased and the number of children (under 18 years of age) living in households increased. Gender, population size of place of residence, education, self-assessments of health, financial situation, respondent or any of his/her family or close friends infected with coronavirus, life satisfaction were not significant predictors of religious commitment decrease (Table 5). The created model was significantly better than a constant only model, χ^2^(10) = 152.788, *p* < 0.001, Nagelkerke *R*^2^ = 0.157.

**Table 5:** The logistic regression analysis predicting decrease in religious commitment based on respondents’ socio-demographic characteristics (N = 1761*).

Predictor	B	SE B	p	Exp(B)
Gender (female-male)	.025	.148	.867	1.025
Age group (increasing)	-.246	.066	**.000**	.782
Population size of place of residence (increasing)	-.023	.052	.661	.977
Education (increasing)	.019	.062	.761	1.019
Number of children (under 18 years of age) living in household (increasing)	.296	.081	**.000**	1.345
Self-assessment of health (increasing)	-.196	.116	.091	1.216
Financial situation (increasing)	.029	.117	.807	.972
Participation in religious practices when not socially isolating (increasing)	-.541	.057	**.000**	.582
Respondent or any of his/her family or close friends infected with coronavirus (no–yes)	-.005	.149	.974	1.005
Life satisfaction (increasing)	-.092	.079	.243	1.097
Constant	-.324	.560	.563	.723

Criterion variable: declared decrease of religious commitment (0 = No, 1 = Yes). Aggregated surveys. Bold indicates were is statistical significance.

* People who chose the answer ‘Not applicable. I do not practice and have not practiced religion’ were excluded from the analysis.

In Table 6, we present how subjective assessment of the change in religious commitment was differentiated by frequency of participation in religious masses, services, and meetings before the pandemic. According to respondents’ declaration, among people who practiced several times a week before the pandemic, the majority (42.6%) experienced an increase in religious commitment during the pandemic. Within this group, however, there were also respondents who experienced a decrease in their religious commitment (15.6%).

**Table 6:** Religiosity before the pandemic and religious commitment during the pandemic (*N* = 1761).

How often do you usually attend masses, services or other religious meetings? (when not socially isolating)	Has your religious commitment changed during the COVID-19 pandemic?				
	Yes, these days I spend more time in prayer, meditation and other religious practices %/(n)	Yes, I spend less time now praying, meditating and other religious practices %/(n)	No, I spend the same amount of time in prayer, meditation and other religious practices %/(n)	Hard to say %/(n)	Not applicable. I do not practice and have not practiced religion %/(n)
Several times a week	42.9% (33)	15.6% (12)	27.3% (21)	11.7% (9)	2.6% (2)
Once a week	14.2% (76)	22.6% (121)	45.5% (244)	17.0% (91)	0.7% (4)
1–2 times a month	4.7% (12)	33.9% (87)	33.9% (87)	24.9% (64)	2.7% (7)
Several times a year	4.4% (20)	9.9% (45)	42.0% (191)	29.5% (134)	14.3% (65)
Once every few years	1.6% (3)	4.3% (8)	23.4% (44)	16.0% (30)	54.8% (103)
Not at all	1.2% (3)	2.8% (7)	6.9% (17)	12.5% (31)	76.6% (190)

* Aggregated surveys. Chi^2^ = 1048.89; df = 20; Cramer’s *V* = 0.386. p<0.001.

The decrease in religious commitment observed during the pandemic was most frequently reported by respondents who attend mass, services or other religious meetings 1–2 times a month, with 33.9% declaring a decline. A decrease in religious commitment was also observed in respondents who attend masses, services or other religious meetings once a week, though most within this group declared that their religious commitment did not change (45.5%).

## Discussion

The global health crisis, caused by the Sars-CoV-2 virus, impacted Polish religiosity. The results of our study clearly indicate that there was deintensification of institutional religion practices in Poland. The respondents’ declarations show that institutional religiosity in Poland during the pandemic decreased almost threefold (from 31.2% to 11.9%). However, as the pandemic continues, the number of people practicing in churches is slowly increasing. Deintensification of institutional religion practices is not unexpected as the pandemic significantly limited participation in institutional religious rituals, through social restrictions aimed at limited the spread of the virus.

During the pandemic, not only institutional ways of practicing faith were more difficult, but also restrictions related to the traditional celebration of holidays and even organization of funerals were introduced. Some of the restrictions on religiosity practice were also dictated by the anti-pandemic policy of other countries where there are traditional places of worship for various religions, such as in Israel where limits prevented pilgrimages to holy places [47]. It is worth emphasizing that the religious restrictions imposed for public health did not arouse any misunderstanding in Poland between the government and religious institutions. However, conflicts and confrontations of this type (explicit or latent) were more significant in Eastern European countries than in Western European countries [48]. Religious communities in Eastern European countries have experienced differently the impact of the pandemic on their functioning and have dealt with the constraints of the pandemic restriction in different ways [47–49].

It is essential to underscore that apart from the restrictions imposed by the government, which resulted in a decline in participation in institutional religious practices, the social control of obligation to attend Sunday mass can be expected to weakened as believers can get dispensation from this duty from the highest Churches’ authorities and were encourage to individual prayer and participation in the mass by media [50]. This sense of responsibility was powerful in Catholicism, particularly in small, local, and rural communities. During the pandemic, church assemblies began to pose a health risk and, in many cases, were indeed a hotspot for the virus [51]. Healthcare experts have warned that traditional religious behavior, such as taking communion, may be spreading the virus [47]. Entering the church at the end of May 2020 raised concerns among 38% of Poles [52]. In this context, limiting institutional religious practices could be an expression of concern for oneself and others and a civic response to the introduced requirements of social distancing. Therefore, due to the pandemic, many religious events were moved to the Internet [32]. However, these actions did not stop the decline in religious involvement, and did not stop the decline in attachment to the local communities. While in 2014 66.0% of Poles declared a relationship with their local community, at the end of 2021 such declarations already constituted 58.0% [53]. Such results are consistent with Sadłoń [52], who claims that the pandemic weakened the behavioral bond of Catholics with their congregation, which plays a vital role in local communities, especially in the countryside. As a consequence of this, it can be speculated that there will be a distancing of people from religion and the Church–especially by those who previously treated Sunday participation in religious practices as an element of tradition and habit.

It can be assumed that during the COVID-19 crisis people will pray more, as supported by both classical [18, 19] and recent literature [20–24]. People who experienced pandemic fear could seek reassurance and support in religion, which is reported as an effective coping strategy in a pandemic [22, 25, 54, 55]. Koenig (2020) claims that emotions experienced in connection with the COVID-19 pandemic may translate into susceptibility to the virus, because fear, stress, and nervousness negatively affect the immune system, but for religious people, prayer can be a good way of reigning over these emotions [22]. Research shows that religious people are better at coping with a variety of diseases [56–58], have less depression [57, 59] and anxiety [60]. As so, it was logical to hypothesize, that Polish society will devote more time to religion during the pandemic, e.g., private prayer even in the case of limitation of institutional religiosity.

Our first research [27], conducted between the 14th and 20th of April 2020, (first wave of the pandemic, when a maximum of 5 people could participate in religious rituals) supported the thesis that in the health crisis of COVID-19 people prayed more. The online survey showed that over one-fifth (21.3%) of the Poles admitted that they devote more time than before the pandemic to prayer and other religious practices. Thus, a significant group of Poles, especially those more religious before the pandemic, found support in religion [27].

However, the study presented in this manuscript does not provide such unequivocal support of this thesis, probably because time has passed, and people tamed the threat. We observed that fewer people increased their religious commitment–measured as a subjective assessment of the time devoted to prayer, meditation and other religious practices (10.6%) than decreased their religious commitment (20.1%). Almost half (43.5%) admitted that their religiosity has not changed, and a quarter (25.8%) were not able to make such an assessment. These data falsified the hypothesis in which we had assumed that it is likely that during the pandemic, more people have increased their religious commitment, expressed in subjective assessment of the amount of time devoted to prayer, meditation and other religious practices than have reduced it, because people in crisis situations seek solace in religion.

To better understand what determines the change in religious commitment, we conducted two logistic regressions to test whether the change could be predicted from socio-demographic characteristics. First logistic regression shows that religious commitment increased as people get older, with younger people, significantly more often than older people, reducing their religious practices during the pandemic. This suggests that the pandemic has deepened the trend of young Poles abandoning religion. The pre-pandemic data of the European Social Survey from 2002–2018 indicated that the decline in declared religiosity in Catholic countries, including Poland, mainly affects young people [12]. Also, an international study by the Pew Research Center indicated that young adults in many countries are less religious than older generations [61]. Of counties surveyed by Pew Research Center, Poland has the largest discrepancy in declared religious importance between those under and those over 40 years [61]. Mendez and Rogaczewska [62] indicate that young people in Poland have been discouraged by the Catholic Church through attempts to influence public life, the conservative social and political interventions. At the same time, Mendez and Rogaczewska believe that young people still have "religious capital"—expressed through various Catholic symbols and the celebration of major Catholic holidays. CBOS data show that decreasing religious practices of young Poles are accompanied by a turn towards left-wing political views [63].

Our first logistic regression shows also that religious commitment increases with the increase in religious practice before the pandemic. This is consistent with changes in religious practice of Poles during the first wave of the pandemic [27]. We have already pointed out that religion, faith and prayer can provide support in difficult times and be a source of hope [23, 54, 64]. The results of our study indicate that religious commitment increased mainly in the group of the most religious Poles (practicing several times a week before the pandemic—42.9%). However, it is also worth mentioning that there were also a small group of people (1.2%) who did not practice at all before the pandemic and they declare an increase in religious commitment. The results of our study are in line with American studies which show that during the first wave of the pandemic, about a quarter of Americans deepened their religious faith, mainly those who often prayed and attended religious meetings before the pandemic [65].

Results of first logistic regression also showed that religious commitment increased when life satisfaction increased, as supported by the scientific literature [66–68]. Other study conducted during the pandemic also found a positive and significant correlation between religiousness and life satisfaction [69].

The second logistic regression showed that religious commitment decreased with age decrease and as the frequency of participation in religious masses, services, and meetings before the pandemic decrease. The decline in religious commitment was mainly by those who participated in masses and other religious rituals once or twice per month before the pandemic. Therefore, even before the introduction of social isolation, their religious commitment was not at the level of every Sunday participation (postulated by the Church).

Religious commitment declined with increasing number of children in the household. This is an interesting result because religious people usually have more children than non-religious people [70, 71]. However, this can be explained by the fact that the pandemic and the accompanying closure of pre-schools, schools, and distance learning put a greater burden on people with children in households, limiting time for religious practices. There are already studies pointing to the problem of the pandemic imposing too many responsibilities on parents, especially mothers [72, 73], and it is reported that women tend to be more religiously committed than men [74, 75].

The pandemic has accelerated the phenomenon of the polarization of Polish religiosity [76–78] as evidenced by the increase in religious commitment during the pandemic by those who previously frequently attended masses and services and those with previous irregular religious practice now spend less time in prayer. Further research could focus on the impact of the intrinsic or extrinsic religious orientation on these processes [79].

Contributing to the decline in religion in Poland has been the publicized cases of pedophilia within the Church [17], and the negligence of the hierarchs. Similarly, the violation of pandemic restrictions and the involvement of hierarchs in the political dispute over tightening the abortion law could have also fostered a decline in religious practice [80].

Based on the results observed in the present study, it can be speculated that the pandemic will accelerate secularization, especially among young people. The processes of privatization of religion and abandonment of institutional religiosity are also likely to intensify. This means that “(…) the social regime related to the epidemic has undoubtedly crushed Polish Catholicism with an additional framework that strengthens the processes observed in Western Europe” [52]. In the face of the threat and uncertainty of the pandemic, some elements of desecularization can be expected, but as the health emergency continues in Poland, they are visible on insignificant level.

After the pandemic, a significant challenge for the Church will be to return to the level of institutional religiosity exhibited before the pandemic. Current trends show that it is unlikely the pandemic will cause a revival of religiosity among Poles since Polish religiosity started to decline before the pandemic due to other factors.

For future studies, qualitative research with people who declared a shift in religious commitment during the pandemic would enable a better understanding the drivers behind it and could explore dimensions of religiosity beyond religious practices. Conducting comparative international studies on the impact of a pandemic on religiosity, taking into account different socio-cultural contexts, would be also very interesting.

The strength of our study was the unique time in which the surveys were conducted—the period of the second and third COVID-19 waves in Poland, i.e., critical moments in many aspects. Public opinion polls in such circumstances make it possible to assess the importance of religion at crisis moments for society, such as while a number of people are fighting for their lives or dying. Such a situation was unprecedented in recent generations in Poland. By aggregation data from two studies the analyzes were based on an exceptionally large sample of respondents (N = 1967), which allowed for more advanced analyzes on the subgroups. Our research may be of interest to researchers from other countries because we describe Polish society, which is still characterized by one of the highest declarations of faith among Catholic countries [12].

The basic study limitation is the quota sample selected from the internet panel. Although this sample reflects the structure of Polish society according to the main socio-demographic characteristics, it does not allow the generalization of the results to all Polish society. The results of CBOS research indicate that approximately 30% of Poles still do not use the Internet regularly, including mainly the elderly and the least educated, i.e., those who are usually more religiously involved [81]. Due to this limitation, we can assume that the number of people attending mass in church, despite the pandemic, and devoting more time to prayer, meditation and other practices in this health crisis, may be slightly underestimated in our research. One potential limitation to consider is that respondents surveyed self-reported so their responses could deviate from their actual behaviors. Another limitation of our study was the simplification of religiosity to religious practices.

## Conclusions

Our research indicates the deintensification of religious practices during the pandemic in Poland, with a threefold reduction in participation in institutional religion. However, as the pandemic continues, a small proportion of people appear to be returning to religious practice. Deintensification of religious practice is also visible in the change the amount of time devoted to prayer, meditation and other religious practices. This means that the crisis, in the long run, did not prompt Poles to seek solace in religion which would be consistent with classical sociological theories [18, 19]. Religious commitment increased in people whose frequency of participation in religious masses, services, and meetings before the pandemic was highest, and decreased in people who practiced not regularly or occasionally. This indicates that the pandemic has accelerated the phenomenon of the polarization of Polish religiosity. Furthermore, we observed a deepening in the secularization of individuals that did not exhibit a strong attachment to the Church and religion before the pandemic and consolidation of religiosity among others that were characterized by above-average religious involvement before the pandemic. These trends require monitoring and further research.

## Supporting information

S1 Appendix Socio-demographic characteristics of the aggregated sample. (DOCX)
