# Supplementary material for: Changes in intensification of religious involvement during the COVID-19 pandemic in Poland
Source: PLoS One. 2022 Jun 15;17(6):e0269015. doi: 10.1371/journal.pone.0269015 (PMC9200353; doi:10.1371/journal.pone.0269015)
Supplement: S1 Appendix — (DOCX) [file pone.0269015.s001.docx]

S1 Appendix

Socio-demographic characteristics of the aggregated sample

| Profile characteristics | *n* | % |
| --- | --- | --- |
| Gender |  |  |
| Female | 1035 | 52.6% |
| Male | 932 | 47.4% |
| Age |  |  |
| 18-29 | 653 | 33.2% |
| 30-39 | 510 | 25.9% |
| 40-49 | 361 | 18.4% |
| 50-59 | 273 | 13.9% |
| 60+ | 170 | 8.6% |
| Education |  |  |
| Primary, lower secondary, vocational | 271 | 13.8% |
| Secondary education | 968 | 49.2% |
| Higher education | 728 | 37.0% |
| Place of residence |  |  |
| Village | 678 | 34.5% |
| City of up to 19,999 | 227 | 11.5% |
| City 20,000 – 199,999 | 407 | 20.7% |
| City 200,000 – 499,999 | 387 | 19.7% |
| City of over 500,000 | 268 | 13.6% |
| Frequency of participation in religious practices |  |  |
| Several times a week | 77 | 3.9% |
| Once a week | 536 | 27.2% |
| 1-2 times a month | 257 | 13.1% |
| Several times a year | 455 | 23.1% |
| Once every few years | 188 | 9.6% |
| Not at all | 454 | 23.1% |
| Financial situation |  |  |
| Very good | 162 | 8.2% |
| Good | 751 | 38.2% |
| Moderate | 912 | 46.4% |
| Poor | 103 | 5.2% |
| Very poor | 39 | 2.0% |
| Self-assessment of health |  |  |
| Very good | 380 | 19.3% |
| Good | 980 | 49.8% |
| Moderate | 525 | 26.7% |
| Bad | 82 | 4.2% |
| Life satisfaction |  |  |
| Satisfied | 1390 | 70.7% |
| Dissatisfied | 402 | 20.4% |
| Hard to say | 175 | 8.9% |
| Political preference |  |  |
| EC Law and Justice – ruling party | 277 | 14.1% |
| EC Civic Coalition –  main opposition | 259 | 13.2% |
| EC Democratic Left Alliance | 182 | 9.3% |
| EC Freedom and Independence Confederation | 193 | 9.8% |
| EC Polish People’s Party | 54 | 2.7% |
| Other | 355 | 18.0% |
| None | 22 | 1.1% |
| Hard to say | 262 | 13.3% |
| Religious commitment during the pandemic |  |  |
| Nowadays I spend more time in prayer, meditation and other religious practices | 147 | 8.3% |
| I spend less time now praying, meditating and other religious practices | 280 | 15.9% |
| I spend the same amount of time in prayer, meditation and other religious practices | 604 | 34.3% |
| Hard to say | 359 | 20.4% |
| Not applicable. I do not practice and have not practiced religion | 371 | 21.1% |
